# Point‐of‐care ultrasound to confirm the position of bronchial blockers in children

**DOI:** 10.1002/jcu.23305

**Published:** 2022-08-30

**Authors:** Junxia Wang, Xin Huang, Weidong Hu, Xianling Cheng, Bin Zhang

**Affiliations:** ^1^ Department of Pediatrics the First Affiliated Hospital of Shandong First Medical University & Shandong Provincial Qianfoshang Hospital Jinan People's Republic of China; ^2^ Department of Biostatistics, School of Public Health, Cheeloo College of Medicine Shandong University Jinan Shandong People's Republic of China; ^3^ Department of Anesthesiology Qilu Children's Hospital of Shandong University Jinan People's Republic of China

**Keywords:** bronchial blocker, children, curtain sign, lung sliding sign, position

## Abstract

**Purpose:**

We described the accuracy of ultrasound in determining the position of bronchial blockers (BBs) in children underwent thoracoscopic surgery.

**Methods:**

We enrolled 52 children with ASA grade I‐III who received thoracoscopic surgery with placement of BBs. Point‐of‐care ultrasound was performed according to the BLUE protocol. The ultrasound‐guided lung sliding sign and curtain sign were used to assess the position of BBs. The accuracy of ultrasound in evaluating the position of BBs, as well as the accuracy and operating time of sliding sign and curtain sign at each examination point were recorded and compared.

**Results:**

The accuracy of ultrasound in evaluating the position of BBs was 88% (46/52, 95% CI 0.69–0.97). When using the curtain sign to assess the position of BBs, the accuracy was 90% (94/104, 95% CI 0.78–0.96), which was significantly higher than when using the sliding sign (65% (136/208), 95% CI 0.55–0.74) (*p* = 0.002). The accuracy of curtain sign at the left mid‐axillary line‐diaphragm and the right mid‐axillary line‐diaphragm was respectively 96% (50/52, 95% CI 0.80–0.99) and 84% (44/52, 95% CI 0.65–0.95), which were higher than that of sliding sign at upper blue points and lower blue points. There was no significant difference in the operating time between two ultrasound signs (the curtain sign, 13.4 ± 8.2 s vs. the lung sliding sign, 16.2 ± 10.0 s, *p* = 0.065).

**Conclusion:**

Point‐of‐care ultrasound can effectively assess the position of BBs. The accuracy of using the curtain sign at the mid‐axillary line‐diaphragm is higher than that of using the lung sliding sign at the anterior chest wall.

## Graphical abstract

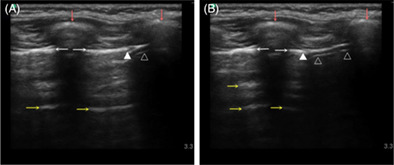

## 1 INTRODUCTION

The bronchial blockers (BBs) can provide a good surgical field for thoracoscopic surgery, and can help reduce the infection of lung tissue on the contralateral side, which is a good airway management tool for one‐lung ventilation during thoracoscopic surgery.^1^, ^2^ The correct placement of the BBs is a key factor for lung ventilation and occlusion during thoracoscopic surgery. Auscultation is a common method to determine the position of BBs.^3^, ^4^ BBs can produce similar lung ventilation to endobronchial intubation. However, auscultation of both lungs cannot fully identify the presence of endobronchial intubation.^5^ Therefore, it is not completely reliable to use auscultation to determine the position of BBs. Fiberoptic bronchoscopy (FOB) is a reliable method to locate the position of BBs.^6^, ^7^ However, it involves invasive procedures that may cause airway infection or injury.^8^, ^9^

Ultrasonography of the lungs is based on the fact that the gas in the lungs creates a huge difference in acoustic impedance to differentiate the lung tissue from the pleura that covers the lung surface. As a result, ultrasound waves are reflected when they reach the pleura, and imaging only shows the pleural layer of healthy lungs. However, when the normal air‐to‐liquid ratio in the lungs is altered by a specific lung disease, especially when the pleura is involved, ultrasound can be locally reflected, creating a unique ultrasound image that helps locate lung lesions.^10^, ^11^ Point‐of‐care ultrasound imaging is a non‐invasive method. Expert consensus and guidelines also recommend point‐of‐care ultrasound for the diagnosis of pediatric pulmonary diseases.^11^, ^12^ The effectiveness of point‐of‐care ultrasound in determining the position of the one‐lung intubation in adults and children has been confirmed in many studies.^5^, ^13^ However, for children, after ultrasound examination, it is still necessary to use other methods to further confirm the intubation position.^14^ Similar to endobronchial intubation, BBs can also produce ultrasound signs, such as the disappearance of the lung sliding sign on the unventilated side.^15^, ^16^ Lung sliding sign is produced by the relative movement of the visceral pleura and parietal pleura during breathing, and is manifested as a faint blinking dynamic sign at or below the pleural line.^17^, ^18^ It weakens or disappears in diseases that can weaken lung movement, such as lung fibrosis, pleural symphysis, and abolition of lung compliance. Many studies have verified the effectiveness of lung sliding sign to distinguish the position of endobronchial intubation.^5^, ^13^ However, the research using lung sliding sign to identify the position of BBs is currently limited to case reports.^15^ Curtain sign is an ultrasound artifact formed by the lower boundary of the lung field in the costophrenic recess with the movement of breathing. It can be used for the diagnosis of lung diseases (such as pleural effusion) and detection of pathological changes in the costophrenic recess (such as atelectasis, consolidations and complete pneumothorax). It is also an ultrasound sign to identify whether the lung tissue is inflated or not,^19^ which also provides a theoretical possibility for the application of the curtain sign to confirm the position of BBs and its occlusion effect. However, there is a lack of research on the use of these two ultrasound signs (lung sliding sign and curtain sign) to determine the position and occlusion effect of BBs as well as on their reliability.

Herein, we investigated the accuracy of using both lung sliding sign and curtain sign in determining the position of BBs in children. Children who received thoracoscopic surgery with placement of BBs were enrolled. Our findings may provide a novel method for confirming the position of BBs.

## 2 MATERIALS AND METHODS

### 2.1 Ethics

The study was approved by the Ethics Committee of our hospital (approval no. ETYY‐2020210) and registered at chictr.org.cn (ChiCTR2000032356). All methods were also performed in accordance with the relevant guidelines and regulations under the committee supervision. Informed consent was obtained from the legal guardians of each subject.

### 2.2 Study subjects

From May 2020 to April 2021, we enrolled children of older than 6 months, with ASA I‐III grade, and undergoing thoracoscopic surgery with BBs placement. Children with diseases that can affect lung sliding sign and curtain sign, such as pleural adhesions, pleural effusion, pulmonary edema, severe lung infection, and airway malformations, were excluded from this study.

### 2.3 Placement of endotracheal tube (ETT) and BBs

The tracheal intubation and BBs placement were performed under general anesthesia. Anesthesia was induced by intravenous bolus of propofol (3 mg/kg) and sufentanil (0.1 μg/kg), and the tracheal intubation and BBs were placed 2 min after the administration of cis‐atracurium (0.2 mg/kg). The sizes of ETT and BBs (5F and 7F, EBT0105, TANPAN, Hangzhou, China) were selected according to the age of the children. The placement method of BBs, that is, intraluminal placement or extraluminal placement, was determined by the anesthesiologists according to their personal experience. For the intraluminal placement, ETT was first placed under the laryngoscope, and then BBs was placed into the trachea through ETT. For extraluminal placement, BBs was first placed into the trachea under laryngoscope, and then ETT was placed into the trachea. After the placement of ETT and BBs, FOB was used to check their position to exclude endobronchial intubation and to guide BBs to be positioned in the target main bronchus. After the BBs balloon was inflated under FOB, the FOB was withdrawn. Children were mechanically ventilated to maintain breathing. The tidal volume was selected according to 6–8 ml/kg, and the respiratory frequency was adjusted to maintain the end‐tidal CO_2_ concentration of 4.0–5.7 Kpa.

### 2.4 Evaluation of BBs position by point‐of‐care ultrasound

**FIGURE 1:**
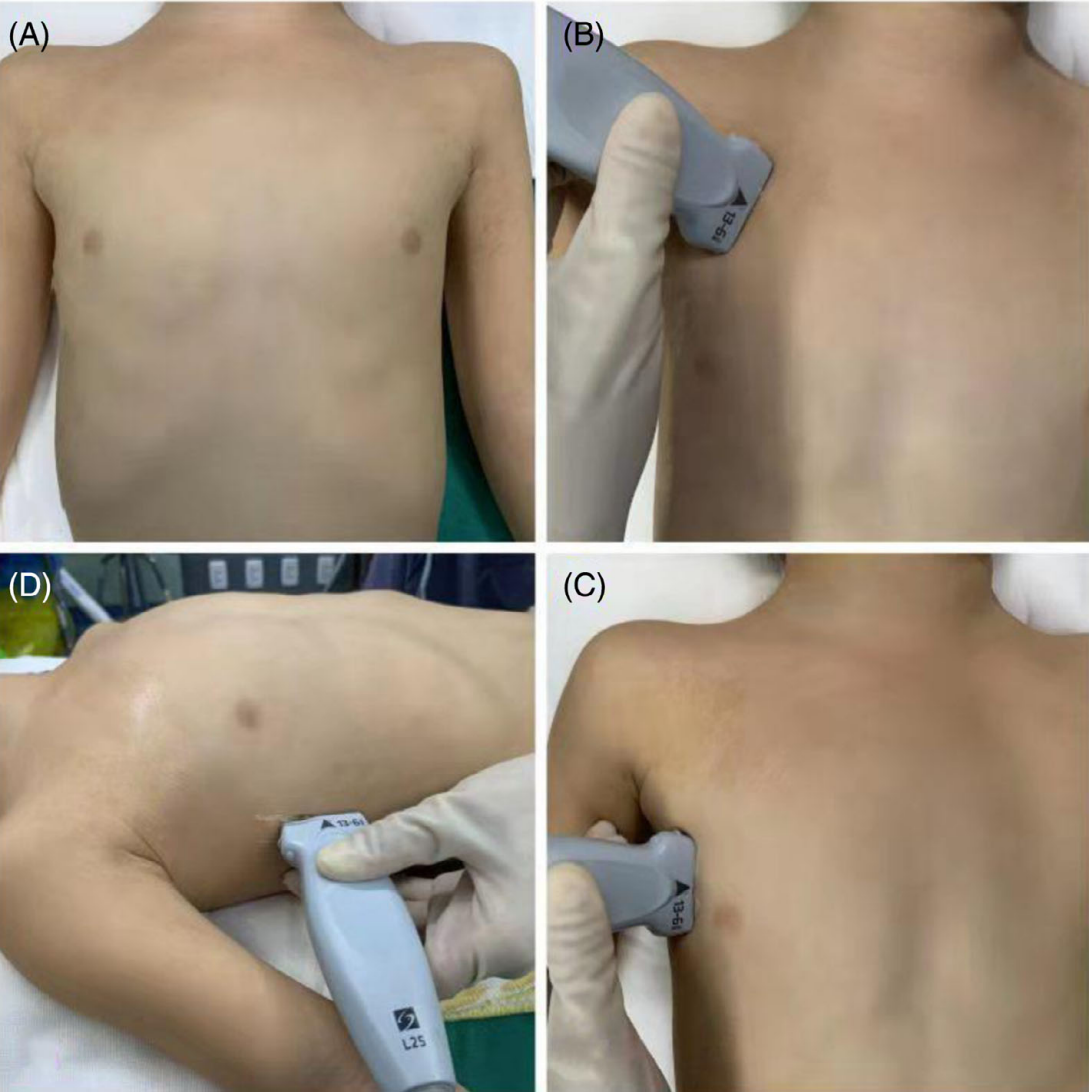
The placement of the probe at each ultrasound examination point. (A) The front of the child's chest; (B) The upper blue point; (C) The lower blue point; (D) The mid‐axillary line‐diaphragm point

**FIGURE 2:**
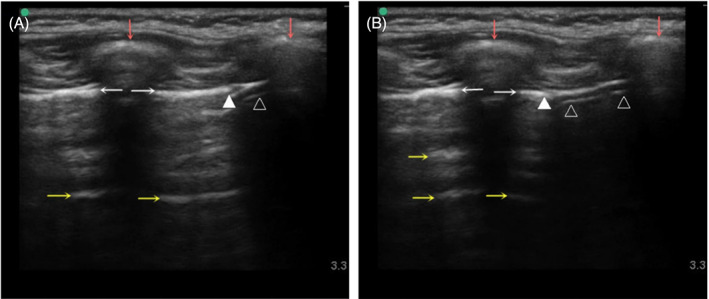
Curtain sign. (A) Ultrasound image at the end of inhalation. (B) Ultrasound image at the end of exhalation. Red arrow: rib; white arrow: pleura; yellow arrow: lung air artifact. Solid arrow: lung base; Open arrow: diaphragm. The overlap of the costophrenic recess onto the abdomen creates a demarcated leading boundary of the lung air artifact. With the breathing movement, the lower boundary of the lung moves back and forth in the cephalo‐sacral direction at the costophrenic recess, giving the impression of a lung curtain

Point‐of‐care ultrasound evaluation was conducted under mechanical ventilation and was performed by one anesthesiologist blinded to BBs placement. The ultrasound examinations of all children were carried out by the same anesthesiologist who had experience in ultrasound examinations on 80 cases. For ultrasound, high frequency linear array probe (13–6 MHz, SonoSite S‐Nerve; SonoSite, Inc., Bothell, WA, USA) was used. The ultrasound images were optimized before examination and the imaging depth was adjusted to be 3–4 times the distance between the A‐lines.^11^ According to the BLUE (bedside lung ultrasound in emergency) protocol, the upper blue points and lower blue points of the bilateral anterior chest wall, and the mid‐axillary line‐diaphragm points where the mid‐axillary line of the bilateral chest wall intersects with the diaphragm were examined.^17^ The order of examination was as follows: the upper blue point on the right, the upper blue point on the left, the lower blue point on the right, the lower blue point on the left, the right mid‐axillary line‐diaphragm point, and the left mid‐axillary line‐diaphragm point. The placement of the probe at each ultrasound examination point is shown Figure 1. On the upper and lower blue points, the BBs position was determined according to the presence or absence of lung sliding sign. If the lung sliding sign disappears, it indicates that the lung on the examination side is not ventilated, that is, the BBs are in the main bronchus of the examination side. If the lung sliding sign is observed, it indicates that the lung on the examination side is ventilated, that is, the BBs are not in the main bronchus of the examination side. On the mid‐axillary line‐diaphragm points, the BBs position was determined based on the presence or absence of curtain signs (Figure 2 and Video S1). If curtain sign is not observed, it indicates that the lung on the examination side is not ventilated and the BBs are in the main bronchus of the examination side. If curtain sign is observed, it indicates that the lung on the examination side is ventilated and the BBs are not in the main bronchus of the examination side. The evaluation results of each ultrasound examination point and the comprehensive evaluation results of the above six examination points were recorded. The operating time at each ultrasound examination point was also recorded.

### 2.5 Reassessment with FOB

After ultrasound evaluation, the anesthesiologist who completed the tracheal intubation performed FOB again to verify the position of BBs, and to check whether BBs are displaced and whether the cuff is fully inflated. According to the results of FOB, the results of ultrasound examination, as well as those of lung sliding sign and curtain sign method in distinguishing the position of BBs, were evaluated.

### 2.6 Statistical analysis

All data were analyzed using SPSS 24.0 software. The measurement data were tested for normality. Data conforming to the normal distribution are represented by the mean ± standard deviation, and compared with t test or ANOVA; while, those not conforming to the normal distribution are represented by the median and interquartile range and analyzed with Wilcoxon rank sum test or Kruskal–Wallis rank sum test. The count data is described by number (%). The unordered classification data was analyzed by *χ* ^2^ test or Fisher's exact probability method, and the unidirectional ordered data was analyzed by rank sum test. The statistically significant difference was considered at *p* < 0.05.

## 3 RESULTS

### 3.1 Basic information of study subjects

A total of 52 children were enrolled in this study, the basic information of the children is shown in Table 1.

**TABLE 1:** Baseline information of study subjects

Subjects characteristics	Patients (*n* = 52)
Age (year)	0.9 (0.7–2.2)
Sex (male/female)	32/20
Height (cm)	74.1 (68.2–88.3)
Weight (kg)	10.0 (9.1–12.8)
Body mass index (kg m^−2^)	16.7 (15.0–18.3)
Diagnosis *n* (%)	
Mediastinum tumor 20 (35.7%)	Congenital cystic adenomatiod malformation 16 (30.7%)
Pulmonary sequestration 12 (23.0%)	Bronchopulmonary dysplasia 4(7.6%)

*Note*: Values are presented as median (inter‐quartile range), or number (percentage) of subjects.

### 3.2 The accuracy of point‐of‐care ultrasound in evaluating the position of BBs

**FIGURE 3:**
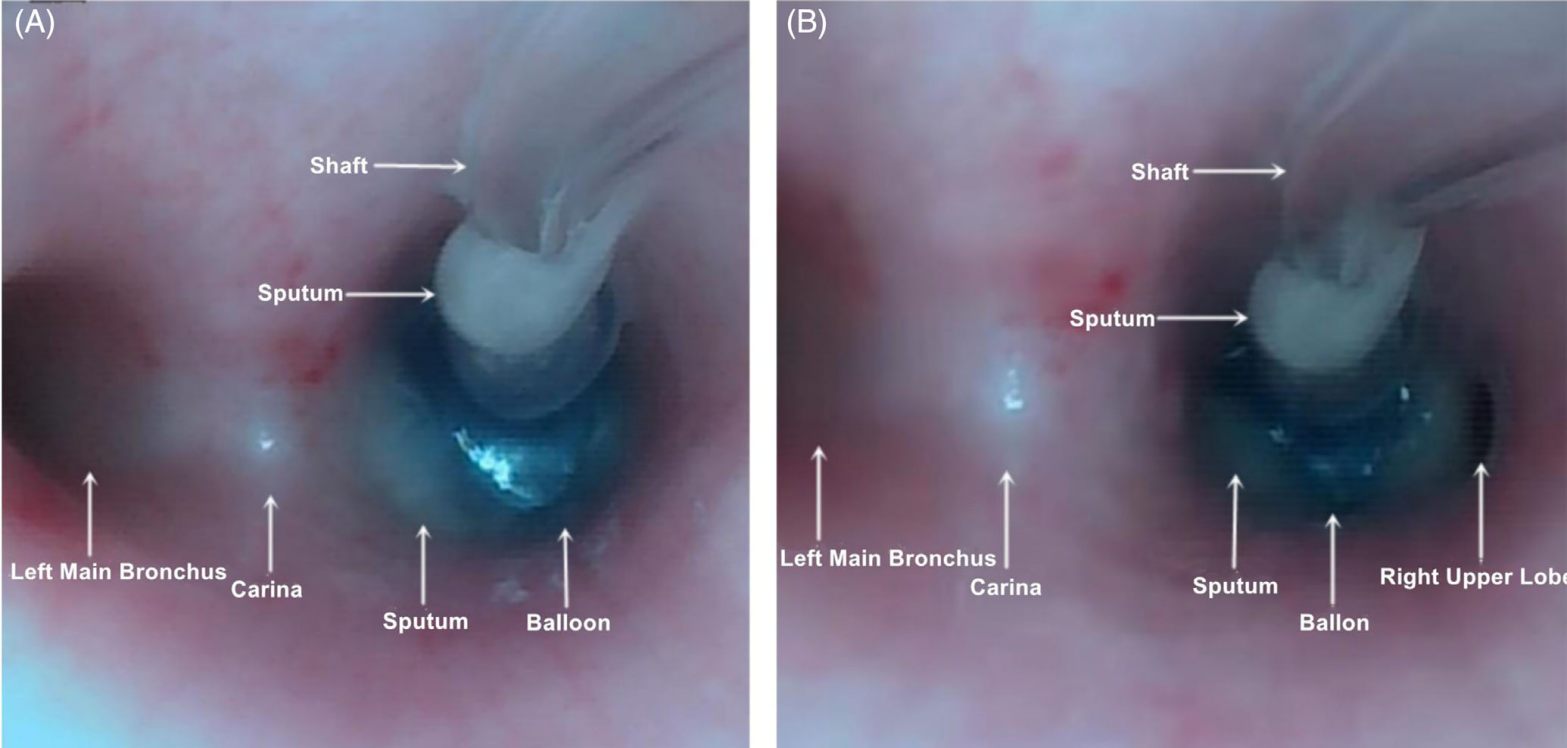
Images under fiberoptic bronchoscopy showing the occlusion of the right lung. (A) The blocker completely blocks the right lung. (B) The upper lobe of the right lung is not completely blocked

FOB reassessment revealed that eight cases of children with BBs placement in the right main bronchus had incomplete occlusion of the upper lobe of the right lung due to cuff displacement or insufficient cuff inflation (Figure 3). Two cases had occlusion of the upper lobe of the left lung because the blocker was inserted too deeply. The ultrasound examination results of these 10 cases were included in this study. There were 4 cases and 12 cases with unclear lung sliding signs at the right lower blue points and the left lower blue points. Thus, the positions of BBs in these cases cannot be determined by using the lung sliding signs.

The accuracy of point‐of‐care ultrasound after comprehensive evaluation was 88% (46/52, 95% CI 0.69–0.97). In the comparison of each ultrasound examination point, the accuracy of using lung sliding sign at the left upper blue point and the right upper blue point were 80% (42/52, 95%CI 0.61–0.93) and 61% (32/52,95% CI 0.40–0.79); and, those at the left lower blue point and the right lower blue point were 61% (32/52, 95%CI 0.40–0.79) and 53% (28/52, 95%CI 0.33–0.73), respectively (Table 2). The curtain sign at the left mid‐axillary line‐diaphragm point and the right mid‐axillary line‐diaphragm point had the highest accuracy, which was 96% (50/52, 95% CI 0.80–0.99) and 84% (44/52, 95% CI 0.65–0.95), respectively. There was significant difference in accuracy among each ultrasound examination point (*p* = 0.001) (Table 2). The overall evaluation accuracy of using the curtain sign was 90% (94/104, 95% CI 0.78–0.96), which was significantly higher than using the lung sliding sign (65% (136/208), 95% CI 0.55–0.74) (*p* = 0.002) (Table 3).

**TABLE 2:** Accuracy at each ultrasound examination point

Right lung	Accuracy (%)	95% CI	Left lung	Accuracy (%)	95% CI	*p*‐value
Upper blue point	61 (32/52)	0.40–0.79	Upper BLUE point	80 (42/52)	0.61–0.93	0.001
Lower blue point	53 (28/52)	0.33–0.73	Lower BLUE point	61 (32/52)	0.40–0.79	
Mid‐axillary‐diaphragm point	84 (44/52)	0.65–0.95	Mid‐axillary‐diaphragm point	96 (50/52)	0.80–0.99	

*Note*: CI: confidence interval. The lung sliding sign was evaluated at the upper and lower blue points. The curtain sign was assessed at the mid‐axillary‐diaphragm points.

**TABLE 3:** Accuracy and examination time of the lung sliding sign and curtain sign

Ultrasound sign	Accuracy (%)	95% CI	*p*‐value	Examination time (s) (Mean ± SD)	*p*‐value
Lung sliding sign	65 (136/208)	0.55–0.74	0.002	16.2 ± 10.0	0.065
Curtain sign	90 (94/104)	0.78–0.96		13.4 ± 8.2	

Abbreviations: CI, confidence interval; SD, standard deviation.

### 3.3 The ultrasound operating time at each examination point

There was no statistically significant difference in the ultrasound operating time at each examination point, as shown in Table 4. There was no significant difference in time between using two ultrasound signs (curtain sign, 13.4 ± 8.2 s vs. lung sliding sign, 16.2 ± 10.0 s, *p* = 0.065) (Table 3).

**TABLE 4:** The examination time at each ultrasound examination point

Right lung	Examination time (s) (Mean ± SD)	Left lung	Examination time(s) (Mean ± SD)	*p*‐value
Upper blue point	18.9 ± 11.0	Upper BLUE point	14.7 ± 9.4	0.094
Lower blue point	13.2 ± 7.9	Lower BLUE point	12.9 ± 8.4	
Mid‐axillary‐diaphragm point	11.9 ± 8.4	Mid‐axillary‐diaphragm point	14.6 ± 7.8	

*Note*: SD: standard deviation. The lung sliding sign was evaluated at the upper and lower blue points. The curtain sign was assessed at the mid‐axillary‐diaphragm points.

## 4 DISCUSSION

Point‐of‐care ultrasound is more and more widely used in anesthesia, which can provide timely diagnosis for anesthesiologists; however, its application in pediatric anesthesia is still less.^20^ In this study, we used two ultrasound signs of lung sliding sign and curtain sign to confirm the position of BBs in children. Six different points on the chest wall were examined. Our results showed that the evaluation accuracy of point‐of‐care ultrasound was 88% (46/52). The accuracy of using the curtain sign at the bilateral mid‐axillary‐diaphragm points was 90% (94/104 47/52), which was significantly higher than that using the lung sliding sign (65% (136/208 68/104)) at upper blue points and lower blue points. There was no significant difference in the operating time at each ultrasound examination point.

Studies have shown that the lung sliding sign has high sensitivity and specificity in evaluating the position of the tracheal intubation and unilateral main bronchial intubation, and its accuracy is greater than that of auscultation.^5^, ^13^ One study in adults have reported that the accuracy of using lung sliding signs to determine endotracheal intubation was 88.7%.^21^ Another study in children have shown that the accuracy of using the lung sliding sign to assess the optimal position of the tracheal tube was 87%.^5^ Similar to one‐lung intubation, BBs can cause the loss of lung activity on the blocked side and produce similar ultrasound signs. In our study, the accuracy of using the lung sliding sign to determine the position of BBs was 65% (136/208), which was significantly lower than the accuracy in previous studies. We suppose that this may be caused by the following reasons. First of all, we used the adult BLUE protocol in selecting the bilateral upper blue points and lower blue points. These four points could avoid the overlap of the lung and the heart in the anatomical position,^22^ which was also the basis for this study to use the adult BLUE protocol. However, the cardio‐thoracic ratio of children is higher than that of adults, and the outer boundary of the heart is closer to the outside of the anterior chest. This caused the inability to evaluate the presence of the lung sliding sign in 16 children in this study (4 cases on the right side and 12 cases on the left side). Secondly, the presence of thymus in children may also interfere with the evaluation of the lung sliding sign of the blue points on both sides. Therefore, we suggest that for children, especially those younger than 1 year old, the more outer part should be selected during the examination of the above hour points, so as to avoid the interference caused by the heart and thymus. In addition, lung pulse, an early ultrasound sign of complete atelectasis,^23^ can interfere with the evaluation of the lung sliding sign. The lung pulse can produce dynamic ultrasound signs at the pleural line that are perpendicular to the pleural line and vibrate in synchronization with the heartbeat when the lung sliding sign disappears. They can be interpreted as the heartbeat transmitted to the pleura formed by the non‐moving lungs.^21^, ^23^ After the disappearance of the lung sliding sign, the dominant lung pulse can produce ultrasound signs similar to the lung sliding sign,^23^, ^24^ which confuses the examiner's judgment. In this study, there were several cases of confusion about the lung pulse and lung sliding signs (data not shown), which led to the failure of evaluation.

The curtain sign can be used to describe the expansion and inflation of the lungs.^19^ In this study, the curtain sign was used for the first time to indirectly evaluate the position of BBs by observing the ventilation of the lungs. Our results found that the accuracy of using the curtain sign to determine the position of BBs was significantly higher than that of using the lung sliding sign. We believe that compared with the weak lung sliding sign at the pleural line, the curtain sign with obvious movement at the costophrenic recess is easier to recognize. It should be pointed out that even if the lung is well blocked with the curtain sign and breath sounds disappear simultaneously, the blocked lung is not completely motionless under ultrasound, but will produce weak movements with the lung ventilation. This may be related to the conduction of pressure in the contralateral thoracic cavity during ventilation (Video S2).

The right main bronchus of children is short, and the displacement of BBs can easily lead to incomplete occlusion of the upper lobe of the right lung.^5^ In addition, insufficient inflation of the BBs cuff can lead to inadequate occlusion of the blocked side and affect the sliding sign and curtain sign. Therefore, it is necessary to compare and comprehensively analyze the ultrasound signs of both lungs. In this study, in eight cases of children with incomplete right upper lobe occlusion, the lung sliding sign at the right upper blue point was present, while that at the right lower blue point disappeared. The curtain sign was present. The lung sliding sign and curtain sign at each point on the left side were present, but the movement of the curtain sign on the right side was weaker than that on the left side. After comparative and comprehensive analysis, we concluded that BBs were in the right main bronchus, but the upper lobe of the right lung was incompletely occluded. In the child with occlusion of the upper lobe of the left lung due to deep insertion of the blocker, there was disappearance of lung sliding sign at the upper blue point on the left, and the movement of the left curtain sign was less than that on the right side. The ultrasound analysis showed that the upper lobe of the left lung was occluded, which was verified by FOB. Studies have pointed out that although the point‐of‐care ultrasound can evaluate segmental ventilation of the lung,^15^, ^25^, ^26^ it cannot clarify the specific lung lobe.^15^ This study demonstrated that the use of ultrasound‐guided curtain sign and lung sliding sign to comprehensively analyze the ventilation of the bilateral lungs can not only evaluate the position of BBs at the lobe level, but also preliminary assess the effect of the occlusion.

For the examination time, we found that the time required for the curtain sign examination was less than that of the lung sliding sign. However, the statistical difference was not significant, which may be related to the relatively small sample size in this study. We believe that finding the moving lower boundary of the lung at the costophrenic recess is the key to the curtain sign. The air movement on the unblocked side makes the lower boundary of the lung easy to identify, and the curtain sign can be recognized in a short time. On the blocked side, there is lack of lung movement, which makes the identification of the lower boundary of the lung difficult, and relatively increases the examination time. There was no difference in the examination time of the lung sliding sign at each point, but for children, the examination time at the lower blue point may take a relatively long time due to the obstruction and interference of the heart.

It is worth noting that although ultrasound can effectively evaluate the position of BBs in a short time, FOB is still an important tool in lung isolation and anesthesia management. First, FOB can directly guide the placement of BBs and observe the position of BBs. Second, FOB examination is rarely affected by the body position of children. In this study, the children maintained the surgical position of contralateral decubitus after completing the placement of BBs in the supine position. Before the operation, the anesthesiologist checked whether the BBS is displaced due to the change of body position using FOB. In the contralateral decubitus position, the ultrasound curtain sign cannot be used to evaluate the ventilation of the bottom lung, which is also a defect of this method. However, this study demonstrates that point‐of‐care ultrasound is also a very helpful method in lung isolation because ultrasound is noninvasive and has reliability in evaluating the position of BBs and in preliminarily evaluating the occlusion effect. In addition, different from the intra‐airway performance of FOB, ultrasound is performed on the body surface of children, which has no effect on ventilation and reduces the incidence of adverse airway events.

This study has some limitations. First, the lung pulse can be presented as intermittent fluctuations at the pleural line synchronized with the heartbeat under the M‐mode method,^23^ which can be used as a basis for identifying lung pulse. We did not use the M‐mode method to distinguish between the lung pulse and the lung sliding signs, which may lead to confusion of the two ultrasound signs and reduced accuracy of the lung sliding sign in evaluating the position of BBs. However, it requires more professional training for the M‐mode method. For beginners, it is easier to observe the pleural sliding caused by lung parenchymal movement.^5^ Secondly, the sample size was relatively small. Further studies with larger sample sizes are warranted.

## 5 CONCLUSION

In short, point‐of‐care ultrasound could be used to evaluate the position of BBs in children with high accuracy. The curtain sign at the mid‐axillary line‐diaphragm examination point, and the lung sliding sign at the upper and lower blue points on the anterior chest wall could be used to assess the position of BBs. Additionally, the accuracy of the curtain sign was higher than that of the lung sliding sign. The accuracy of ultrasound evaluation at multiple points was higher than that at single point. It is recommended to conduct comprehensive evaluation of the lungs on both sides to improve the accuracy of ultrasound evaluation, and to conduct a preliminary evaluation of the occlusion effect.

## FUNDING INFORMATION

This study was supported by the Comfortable Medical Fund of Shandong Medical Association.

## CONFLICT OF INTEREST

The authors report no conflict of interest.

## ETHICS STATEMENT

The study was approved by the Ethics Committee of Qilu Children's Hospital of Shandong University (approval no. ETYY‐2020210) and registered at chictr.org.cn (ChiCTR2000032356). All methods were also performed in accordance with the relevant guidelines and regulations under the committee supervision.

## PATIENT CONSENT STATEMENT

Informed consent was obtained from the legal guardians of each subject.

## Supporting information


**Video S1** The video showing the lung curtain sign.

**Video S2** The conduction of pressure in the contralateral thoracic cavity during ventilation.

## Data Availability

The data that support the findings of this study are available on request from the corresponding author. The data are not publicly available due to privacy or ethical restrictions.
